# A case study on the dermatoscopic findings of Darier's disease in skin type VI

**DOI:** 10.1002/ski2.282

**Published:** 2023-09-01

**Authors:** Samavia Khan, Banu Farabi, Alina Zufall, Anjelica Peacock, Kenneth Helmandollar, Kenneth Shulman, Bijan Safai

**Affiliations:** ^1^ Center for Dermatology Rutgers Robert Wood Johnson Medical School Somerset New Jersey USA; ^2^ Dermatology Department New York Medical College Valhalla New York USA; ^3^ Dermatology Department NYC Health + Hospital/Metropolitan New York New York USA; ^4^ Dermatology Department NYC Health + Hospital/Coney Island Brooklyn New York USA; ^5^ Dermpath Diagnostics White Plains New York USA

## Abstract

We present dermatoscopic findings of long‐standing, untreated Darier's disease (DD) in skin type VI that differs from current findings in literature. Robust hyperkeratotic polygonal‐shaped plugs without a surrounding white halo and classic vascular features were noted on the anterior scalp, neck, axilla, midline trunk, and extensors. Through this case, we aim to contribute to emerging literature in describing features of DD under dermatoscopy to augment diagnosis.

## Graphical abstract

1. Untreated, long‐standing Darier's disease (DD) may obscure characteristic dermatoscopic findings of acanthosis (white halo) and linear/dotted vessels (vascular features). 2. Untreated DD may present with robust hyperkeratotic polygonal‐shaped papules coalescing into plaques. 3. It is noteworthy to recognise these unique dermatoscopic findings of DD to guide diagnosis and differentiation from other acantholytic and dyskeratotic disorders.

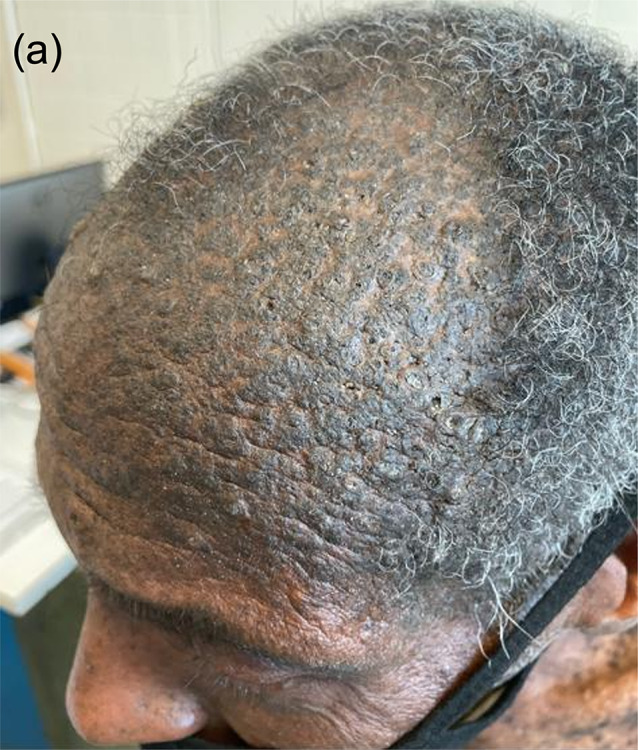

## 1 INTRODUCTION

Dermatoscopy has shown value as an ancillary tool in diagnosing acantholytic and dyskeratotic disorders. Darier's disease (DD), also known as keratosis follicularis, is a rare autosomal dominant acantholytic dermatosis. It presents as a persistent eruption of hyperkeratotic papules and/or plaques in a seborrhoeic distribution, sometimes with nail and mucosal involvement. In a case series of 11 patients with biopsy‐proven‐DD, 100% of patients demonstrated a central yellow‐brown area (polygonal or star‐like) with a surrounding white halo.^1^ Pinkish homogeneous structureless background was also seen in 100% of patients (*n* = 11).^1^ Dotted and/or linear vessels were noted in 63.8% of patients (*n* = 7).^1^ Whitish scale surrounding polygonal brown‐yellow areas were seen in 27.3% of patients (*n* = 3).^1^ Here, we report a case of biopsy‐proven DD which presented with robust hyperkeratotic lesions in which the classic dermoscopic findings of a white‐surrounding halo^1^, ^2^, ^3^ and dotted and/or linear vessels^1^, ^2^, ^3^ was not visible.

## 2 CASE

A 59‐year‐old male patient, Fitzpatrick skin type VI, was referred to the dermatology clinic for pruritic, dark bumps on the skin that were present since childhood. Minimal improvement was achieved using ammonium lactate 12% cream twice daily for 8 months. The patient had a family history of similar lesions in his mother and sister, however, the lesions were never diagnosed.

**FIGURE 1:**
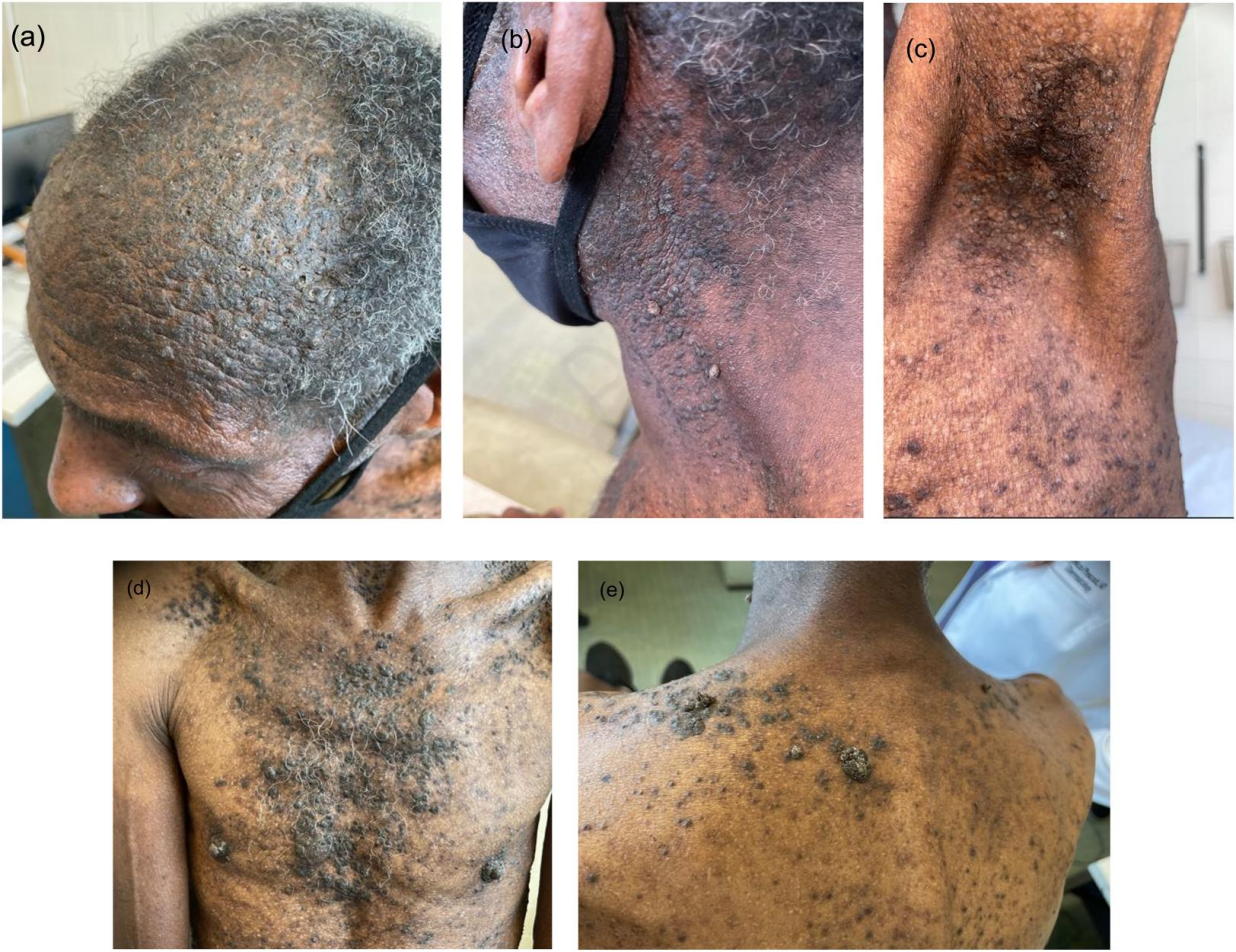
Clinical images of Darier’s disease in a 59‐year‐old male with long‐standing history of extensive hyperkeratotic papules. Multiple, dark, brown‐coloured greasy and scaly papules located on the scalp (a), lateral neck (b), axillae (c), and trunk (d/e) (anterior/posterior). Some lesions demonstrate a warty appearance with coalescing small papular lesions on the axillae and posterior trunk (b, e).

**FIGURE 2:**
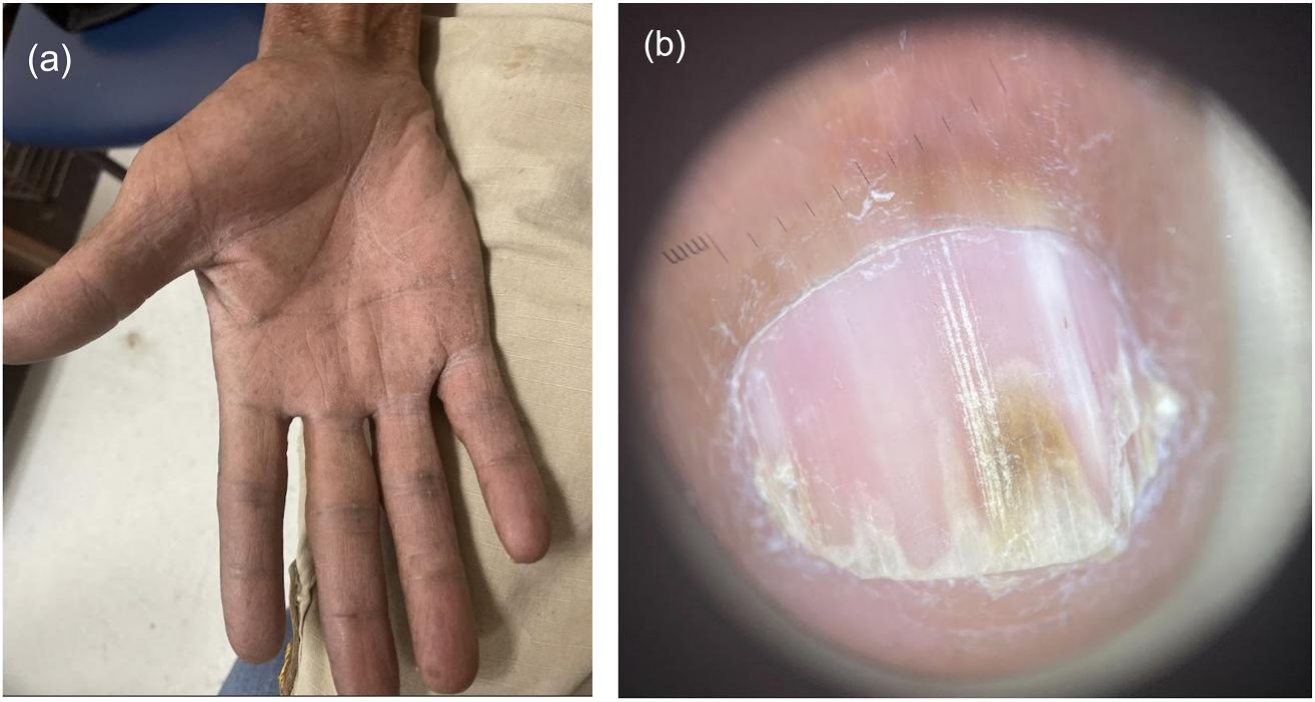
Clinical hand and nail images of Darier’s disease in a 59‐year‐old male. (a) Palmar hyperlinearity with pitting of the right hand of the patient. (b) Dermatoscopic image of right thumb shows periungual scaling, longitudinal leukonychia, yellow discolouration of the distal nail plate due to onycholysis and V‐shaped nicking on the distal edges.

Physical examination revealed hundreds of dark brown, 2–5 mm, polymorphous, hyperkeratotic papules widely distributed on the frontotemporal scalp, lateral neck, axillae, and trunk (Figure 1a–e). Some papules exhibited white scale and a papillomatous and/or verruciform appearance. Some smaller papules were seen coalescing into larger plaques. The patient had palmar pitting and distal nail splitting with longitudinal leukonychia (Figure 2a,b).

**FIGURE 3:**
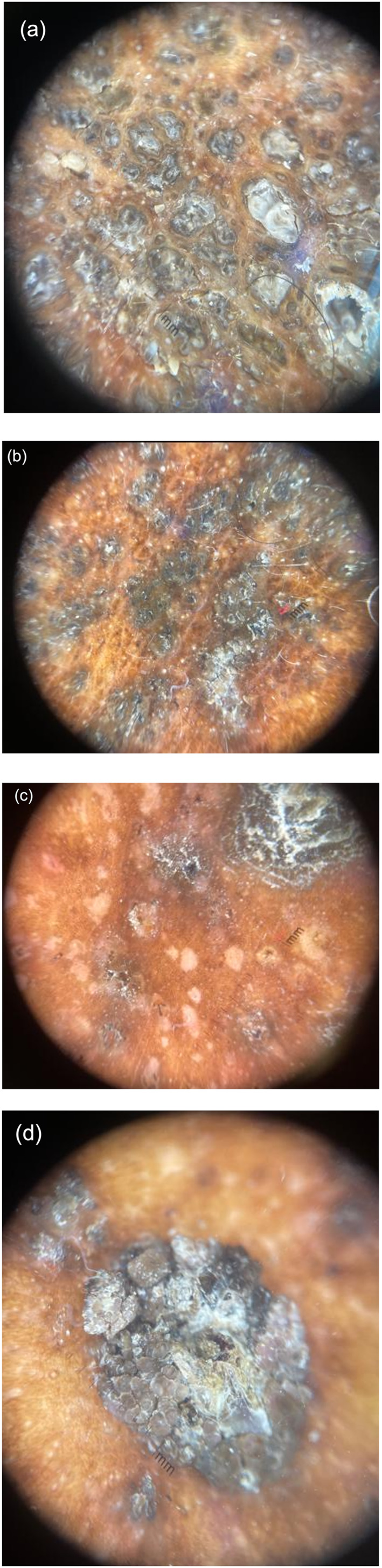
Dermatoscopic images of Darier's disease. (a) Scalp lesions show dilated polygonal openings with raised borders and a central brown‐whitish hyperkeratotic plug. (b) Lesions from the left lateral neck show brown papules with white scale coalescing into plaques. (c) Lesions from the anterior tibia show hyperkeratotic brown papules in addition to hypopigmented macules. (d) Lesion on the posterior trunk shows hyperkeratosis and papillomatous surface with a keratin plug.

Dermatoscopic examination of the scalp revealed dilated polygonal openings with raised borders and a central brown‐whitish hyperkeratotic plug (Figure 3a). Lesions from the left lateral neck show brown papules with white scale coalescing into plaques (Figure 3b). Lesions from the anterior tibia show hyperkeratotic brown papules in addition to hypopigmented macules (Figure 3c). Lesion on the posterior trunk shows hyperkeratosis and papillomatous surface with a keratin plug (Figure 3d). Differential diagnoses included DD, folliculotropic mycosis fungoides, vitamin A deficiency (phrynoderma), and epidermodysplasia dysplasia verruciformis. Biopsy showed focal acantholytic dyskeratosis consistent with DD. The patient opted to proceed with Isotretinoin 20 mg twice daily (with routine blood monitoring) for 5 months and his lesions, especially of the forehead, trunk, and axillary areas, flattened and were followed to evaluate for complete resolution.

## 3 DISCUSSION

DD shows three zones on dermoscopy: ‘central light brown follicular opening, surrounding dark brown structure, and peripheral whitish halo’.^4^ We present a case in which the robust hyperkeratotic spikes of untreated, biopsy‐proven DD obscured the classic acantholysis (white halo) and vascular features. In addition, we suspect that the patient's skin type may have further contributed to clinical and dermoscopic findings that appear different than those reported in the literature. Given the polymorphous nature of the lesions, the differential diagnosis was broad. Folliculotropic mycosis fungoides and phrynoderma present with generalised follicular hyperkeratotic papules both clinically and dermatoscopically. Because of the monomorphic nature of phrynoderma and the patient's polymorphic lesions, as well as the patient's lack of malabsorption history common with phrynoderma, this diagnosis was lower on the differential. Given the verruciform appearance of lesions, epidermodysplasia verruciformis was considered. However, epidermodysplasia verruciformis is autosomal recessive, unlike DD, which is autosomal dominant and present in every generation and the patient did not have a history of nonmelanoma skin cancer. Given the patient's family history of his mother having similar lesions, lesions being present since childhood, and palmoplantar pitting and nail findings, DD was yielded as a diagnosis and confirmed with histology. In the presence of follicular hyperkeratosis with polygonal edges, DD should be kept in mind as a differential diagnosis, especially in the presence of positive family history and clinical features.

Through this case, we aim to share a unique dermatoscopic presentation of untreated DD, which lacks the classic white halo and the vascular pattern. When presented with extensive polymorphous lesions in untreated, darker skin type patients, dermatoscopy presents as a useful complementary tool to distinguish DD from other differentials.

## AUTHOR CONTRIBUTIONS

**Samavia Khan**: Conceptualization (equal); data curation (equal); formal analysis (equal); investigation (equal); writing—original draft (equal); writing—review & editing (equal). **Banu Farabi**: Conceptualization (equal); investigation (equal); supervision (equal); writing—original draft (equal); writing—review & editing (equal). **Alina Zufall**: Conceptualization (equal); investigation (equal); methodology (equal); resources (equal); writing—original draft (equal). **Anjelica Peacock**: Conceptualization (equal); writing—original draft (equal). **Kenneth Helmandollar**: Conceptualization (equal); writing—original draft (equal). **Bijan Safai**: Conceptualization (equal); writing—original draft (equal).

## CONFLICT OF INTEREST STATEMENT

The authors declare they have no conflicts of interest.

## ETHICS STATEMENT

The patient gave written consent for photographs and medical information to be published in print and online and with the understanding that this information may be publicly available.

## Data Availability

Data sharing not applicable to this article as no datasets were generated or analysed during the current study.
